# RNA-seq analysis reveals extensive transcriptional plasticity to temperature stress in a freshwater fish species

**DOI:** 10.1186/1471-2164-14-375

**Published:** 2013-06-05

**Authors:** Steve Smith, Louis Bernatchez, Luciano B Beheregaray

**Affiliations:** 1 Molecular Ecology Laboratory, School of Biological Sciences, Flinders University, Adelaide, SA 5001, Australia; 2 Institut de Biologie Intégrative et des Systèmes, Université Laval, Québec, QC G1V 0A6, Canada; 3 Current address: Department für Integrative Biologie und Evolution, Veterinärmedizinische Universität Wien, Vienna 1160, Austria

**Keywords:** Rainbowfish, *Melanotaenia duboulayi*, Transcriptomes, Climate change, Thermal adaptation

## Abstract

**Background:**

Identifying genes of adaptive significance in a changing environment is a major focus of ecological genomics. Such efforts were restricted, until recently, to researchers studying a small group of model organisms or closely related taxa. With the advent of next generation sequencing (NGS), genomes and transcriptomes of virtually any species are now available for studies of adaptive evolution. We experimentally manipulated temperature conditions for two groups of crimson spotted rainbowfish (*Melanotaenia duboulayi*) and measured differences in RNA transcription between them. This non-migratory species is found across a latitudinal thermal gradient in eastern Australia and is predicted to be negatively impacted by ongoing environmental and climatic change.

**Results:**

Using next generation RNA-seq technologies on an Illumina HiSeq2000 platform, we assembled a *de novo* transcriptome and tested for differential expression across the treatment groups. Quality of the assembly was high with a N50 length of 1856 bases. Of the 107,749 assembled contigs, we identified 4251 that were differentially expressed according to a consensus of four different mapping and significance testing approaches. Once duplicate isoforms were removed, we were able to annotate 614 up-regulated transfrags and 349 that showed reduced expression in the higher temperature group.

**Conclusions:**

Annotated blast matches reveal that differentially expressed genes correspond to critical metabolic pathways previously shown to be important for temperature tolerance in other fish species. Our results indicate that rainbowfish exhibit predictable plastic regulatory responses to temperature stress and the genes we identified provide excellent candidates for further investigations of population adaptation to increasing temperatures.

## Background

The ability of species and populations to adapt to environmental change is the cornerstone of the emerging field of ecological genomics [1,2]. Until recently, genome-wide studies of genetic adaptation in non-model organisms were not possible. With the advent of massively parallel next generation sequencing technologies (NGS), these types of studies have become a reality [3] and while many of the challenges and preferred strategies are still being addressed [4-6], empirical studies are now starting to be reported [7-14]. Studies of transcriptome level responses to environmental change offer an opportunity to understand the underlying genetic basis for adaptation. Such studies represent a powerful approach to assessing the genes involved in adaptation to a changing climate, particularly increasing temperatures. By profiling transcriptional changes induced by temperature stress, it is possible to identify the gene regions or pathways that are likely to be the targets of selection. This information is crucial to enable researchers to assess levels of variation across these gene regions, at a landscape scale, to predict the capacity of organisms to adapt to a warming climate.

Genes involved in physiological adaptation to temperature stress have been uncovered in many species. Heat shock proteins [15], alcohol dehydrogenase [16] and lactate dehydrogenase genes [17] have all been shown to be related to heat tolerance. In fish, the list of candidates also includes many from other gene regions related to respiration and protein binding [18-20]. Apart from differences in coding regions, transcriptional regulation is also a source of variation that can potentially contribute to adaptive evolutionary change, particularly in the early stages of divergence. Studies in natural populations of gobies (*Gillichthys mirabilis*) have shown that short term exposure (<8 hours) to a temperature of 32°C induces a strong upregulation of heat shock proteins (Hsps) in both gill and muscle tissues [21]. Many other transcripts related to a wide variety of biological processes including protein homeostasis, cell cycle control, cytoskeletal reorganisations, metabolic regulation, and signal transduction were differentially expressed in treatment and control groups. The majority of these genes displayed tissue-specific responses presumably related to the differing molecular functions associated with each tissue type. Logan and Somero [22] found that, with long-term acclimation to increased temperature (up to 28°C), there was no upregulation of stress-related proteins and only slight, although detectable, differences in expression of genes involved in protein biosynthesis, transport and various metabolic categories. This they suggest indicates evidence of long-term acclimation showing a steady state condition involving relative energy costs for different processes. They later showed however, that stress related genes (*HSP70, UBIQ*, and *CDKN1B*) were induced in long-term acclimatised fish subsequently exposed to acute heating conditions (4°C/hour) and that the onset temperature for significant expression change varied according to acclimation temperature [23]. Quinn *et al.*[24] also found increased expression of HSPs and Ubiquitin in Arctic charr (*Salvelinus alpinus*) exposed to temperature stress and reported a down regulation of haemoglobin genes in fish that showed tolerance to increased temperatures. Another cold climate fish, *Trematomus bernacchii,* has been shown to be unable to mount a heat shock response despite retaining the heat shock gene *Hsp70* and the regulation factor HSF1 [25]. Further work showed that many other genes associated with the cellular stress response were induced by heat stress. The inability to mount a heat shock response however, highlights the susceptibility of this species to global warming and raises the question as to how this and other species will be able to adapt to increasing temperatures.

Buckley and Hofmann [26] examined the extensive plasticity in Hsp induction in gobies acclimatised to different thermal backgrounds (13°C, 21°C, and 28°C). They found that the activation temperature of the transcriptional regulator HSF1 was positively associated with the acclimatisation temperature indicating that plasticity in heat shock response is linked to plasticity in the regulatory framework governing Hsps. While adaptive plasticity is often seen as a mechanism that can slow or dampen divergent selection, it has been argued that it can also lead to rapid speciation if there are strong correlations between phenotype and environment combined with significant population structure [27]. By examining the transcriptomic response to temperature stress we can develop a better understanding of the genes and biochemical pathways that are fundamental to physiological acclimatisation to a warming environment and gain insights into the regulatory changes that accompany adaptation over evolutionary timescales [28].

Australian rainbowfish are an ideal species group to test hypotheses about the genetic responses to increasing temperatures. In particular, the crimson-spotted rainbowfish (*Melanotaenia duboulayi*) is a subtropical freshwater species found along a north–south temperature gradient in eastern Australia. Their distribution ranges over several ecoregions which, coupled with a strong population structure and local abundance [29-31], makes them a well suited model for studying local adaptation. The ease of maintaining captive populations of rainbowfish also make them amenable to a range of laboratory-based experimental studies [32-34]. In this study, we maintained groups of *M. duboulayi* at ambient and elevated temperature levels and then used an RNA-seq approach to assess transcriptome level changes related to temperature stress. Our aim is to provide an initial investigation of the transcriptomic response to thermal stress in rainbowfish. As such, this will allow for the screening of many more individuals via genotyping of candidate SNPs. In addition we present the first annotated transcriptome and gene catalogue for the order Atheriniformes. Our goal is to identify key candidate genes and make a first step towards understanding the important biochemical pathways on which selection is likely to act in a warming climate.

## Methods

### Source of fish and design of temperature trial

Crimson spotted rainbowfish were collected using a hand-net from a location in the upper reaches of the Brisbane River, near the township of Fernvale (27°26'37.39"S, 152°40'12.76"E). Water monitoring data from the Queensland Department of Environment and Resource Monitoring (DERM) show the average daily mean temperatures for this location ranged between 12.2°C in winter and 28.3°C in summer from January 1^st^ 2004 to January 1^st^ 2011 (http://watermonitoring.derm.qld.gov.au). Fish were transported live to Flinders University animal rearing facility and acclimatised at a temperature of 21°C for a period of 30 days prior to the start of the temperature trials. For the trials we used only adult male rainbowfish of about the same length (a proxy for age), since gender and age can affect expression responses [35]. These individuals were randomly assigned to a treatment or a control group (n = 6 per group). Temperature in the treatment group was increased by 2°C per day over a period of six days towards a target of 33°C. This target represents the projected average summer temperature for this region in 2070 based on a high emission scenario of the International Panel on Climate Change: http://www.climatechangeinaustralia.gov.au/qldtemp15.php. This temperature condition was then maintained for 14 days. The control group was kept at 21°C for the duration of the experiment. All animal handling was performed in accordance with the Australian Code of Practice for the Care and Use of Animals for Scientific Purposes, 2004 and approved by the Flinders University Animal Welfare Committee (AWC E342).

### RNA extraction, Illumina library preparation and sequencing

Upon completion of the temperature trial, fish were sacrificed using AQUI-S® solution [36] and dissected immediately to remove their livers. Although increased temperature has been shown to differentially induce expression changes in different tissue types [21,37], we were restricted to examining just one tissue type due to logistical constraints. We selected liver due to previous research linking this tissue type to heat stress responses [38-40]. Total RNAs were individually extracted using the Ambion Magmax™-96 total RNA isolation kit (Life Sciences) according to the manufacturer’s instructions. Briefly, 5 mg of tissue was placed in the lysis solution and homogenised in Qiagen Tissuelyzer™ for a period of 30 sec. Nucleic acids were captured onto magnetic beads, washed and treated with DNase. Total RNA was then eluted in 50 μl elution buffer. RNA quality and concentration was measured using an RNA Pico chip on an Agilent Bioanalyzer. Normalised starting quantities of total RNA were then used to prepare 12 separate Illumina sequencing libraries with the TruSeq™ RNA sample preparation kit (Illumina). Library preparation was performed as per the manufacturer’s instructions. In the final step before sequencing, all 12 individual libraries were normalised and pooled together using the adapter indices supplied by the manufacturer (Illumina MID tags 2, 4–7, 12–16, 18, 19). Pooled sequencing was then performed as 101 bp, paired-end reads in a single lane of an Illumina HiSeq2000 instrument housed at the Ramaciotti Centre for Gene Function Analysis, University of New South Wales.

### Quality control and de novo assembly

Sequence data were sorted by individual and adapters were trimmed by the service provider prior to analysis. Quality filtering was performed using the FastX-toolkit suite of pre-processing tools (http://hannonlab.cshl.edu/fastx_toolkit/index.html) in a Galaxy setting [41]. Based on the FastX quality statistics, the first two and last 5 bases were trimmed from each read as they had consistently low phred scores (<Q15). Paired reads were then joined and a quality filter applied such that any combined reads having <90% of bases with a phred score of Q20 or higher were discarded. Paired reads were then split and interleaved to suit the input style of the *de novo* assembly program. Transcriptome assembly was performed *de novo* with the program Velvet/Oases [42]. This program reconstructs independent assemblies based on different k-mer values used to build a de Bruijn graph. The program then uses dynamic error removal adapted to RNA-seq data and implements a robust scaffolding method to predict full length transfrags. Multiple single k-mer assemblies are then merged to cover genes at different expression levels without redundancy. Two individuals from each of the treatment and control groups were pooled as input for the assembly. Assemblies were compiled for a k-mer range of 19 to 49 with an expected insert size between paired ends of 300 bp and a coverage cut-off value set to 4.2. We tested different merged assembly ranges based on the summary statistics for each individual k-mer assembly [43]. The outcome of each merge was assessed with respect to the optimal assembly parameters [4]. The optimal assembly should achieve the best balance between large median, mean and N50 contig lengths while minimising the total number of contigs but maintaining a large summed contig length. As Oases is vulnerable to mis-assembly at low k-mer values, we adopted a conservative approach of merging k-mer values > k = 19. Optimal assembly was achieved with a k-mer range of 19 to 41.

### Mapping of sequence reads and differential expression analysis

To test for differential expression (DE), individual sequence reads for each sample were mapped back to the assembled transcriptome with the alignment program Bowtie [44]. Bowtie was implemented in the –v alignment mode with the maximum number of mismatches set to 3. Paired end reads were aligned to the transcriptome with both read pairs needing a valid alignment within a given locus to be counted as a match. If more than one alignment was possible the best match was reported according to the least number of mismatches for each read and overall for the pair. The reproducibility of the alignment approach was tested by performing the mapping step with BWA, an alternative alignment program [45]. The number of reads aligning to each transfrag for each sample was calculated with the IdxStats command of Samtools [46]. Count data was then used as input for the program DESeq [47] which estimates variance-mean dependence in the data and tests for differential expression based on the negative binomial distribution. The six samples from each treatment were used to generate mean expression levels with associated variances. Differential expression was tested at a significance level of α= 0.05 adjusted to match a 5% false discovery rate using the Benjamini-Hochberg procedure. The threshold for fold-change differences is determined by the significance testing as the power to detect significant differential expression depends on the expression strength. For weakly expressed genes, stronger changes are required for the gene to be called significantly expressed. We also compared DE methodology by running the EdgeR program to assess significant differences in the count data. A consensus list of DE genes was then generated from the four analysis approaches adopted (i.e. Bowtie-DESeq, Bowtie-EdgeR, BWA-DESeq, BWA-EdgeR). Significantly up and down regulated transfrags were selected and blasted against the NCBI database using blastx in the program Blast2GO [48]. Blastx was performed against the NCBI nucleotide database with the minimum E-value score set to 1.0E-06. To assign gene ontology terms to each annotated sequence, successful blast hits were mapped and annotated using Blast2GO for the entire assembled transcriptome with the annotation cut-off threshold set to 55 and the GO level weighting set to 5.

## Results and discussion

### Raw sequencing data and quality statistics

The single lane of Illumina HiSeq2000 produced close to 128 million paired-end reads (2 × 101 bp). After trimming and quality filtering, 12.3% of reads were discarded leaving over 224 million reads for downstream analysis (2 × 94 bp). The final number of reads per individual ranged from 11.7 million to 29 million (mean = 18.7 million ± 1.4 million). The number of reads in each treatment group was well balanced with 112.3 million in the 21°C group and 112.0 million in the 33°C group (Additional file 1: Table S1). We selected the best k-mer merge range for assembly based on the distribution of assembly statistics for the individual k-mer assemblies from k = 19 to k = 49 (see Table 1). The merged assembly from a k-mer range of 21 to 39 scored best on the balance of these parameters with a N50 value of 1,856 and a total number of contigs of 107,749. While this range may exclude some rare, low-abundant transcripts, it presents a more conservative and reliable approach to differential expression testing by emphasising the accuracy of the assembly rather than the identification of low-abundant transcripts from both treatments. Annotation of the transfrags with the Blast2Go software suite resulted in 65,105 (60.4%) blast hits and 53,278 (49.4%) successfully annotated sequences.

**Table 1:** **Assembly statistics for k-mer lengths 19–49 and different k-mer merge ranges from the Oases *de novo *assembly program**

	**k19**	**k21**	**k23**	**k25**	**k27**	**k29**	**k31**	**k33**	**k35**	**k37**	**k39**	**k41**	**k43**	**k45**	**k47**	**k49**	**k19_39**	**k21_39**	**k25_47**	**k21_49**
Total sequences	1.2E+05	7.3E+04	6.2E+04	5.5E+04	5.2E+04	5.0E+04	4.8E+04	7.0E+04	8.2E+04	8.1E+04	8.2E+04	4.3E+04	4.1E+04	4.0E+04	3.9E+04	5.6E+04	4.5E+05	1.1E+05	4.0E+05	4.9E+05
Total bases	6.7E+07	6.1E+07	5.6E+07	5.3E+07	5.1E+07	5.0E+07	4.9E+07	6.2E+07	7.2E+07	7.3E+07	7.2E+07	4.4E+07	4.3E+07	4.2E+07	4.1E+07	5.1E+07	4.8E+08	1.3E+08	4.4E+08	5.5E+08
Min sequence length	7.1E+01	1.0E+02	8.1E+01	1.0E+02	9.8E+01	1.0E+02	9.0E+01	1.0E+02	1.0E+02	1.0E+02	1.0E+02	9.9E+01	1.0E+02	1.0E+02	1.0E+02	1.0E+02	1.0E+02	1.0E+02	1.0E+02	1.0E+02
Max sequence length	1.5E+04	1.7E+04	2.0E+04	1.8E+04	2.1E+04	2.3E+04	1.8E+04	1.2E+04	1.3E+04	1.3E+04	1.4E+04	1.7E+04	1.7E+04	1.7E+04	1.7E+04	1.4E+04	2.3E+04	1.7E+04	2.3E+04	2.3E+04
Average sequence length	558.04	837.19	906.27	960.63	979.44	991.21	1010.47	884.15	888.33	901.83	884.62	1026.19	1037.21	1042.06	1049.7	903.57	1071.74	1245.3	1114.01	1124.62
Median sequence length	356	527	546	580	584	590	605	595	583	584	568	624	634	639	650	608	780	930	805	818
N50 length	873	1397	1585	1686	1746	1759	1801	1398	1460	1493	1492	1785	1789	1795	1786	1457	1589	1856	1671	1689
(A + T)s	55.25%	55.32%	55.25%	55.16%	55.16%	55.17%	55.12%	55.07%	55.21%	55.35%	55.38%	55.04%	55.03%	55.05%	55.03%	54.85%	55.95%	55.11%	55.69%	56.13%
(G + C)s	43.99%	44.27%	44.47%	44.60%	44.64%	44.67%	44.72%	44.83%	44.63%	44.49%	44.48%	44.86%	44.87%	44.87%	44.89%	45.08%	44.05%	44.89%	44.31%	43.87%
Ns	0.77%	0.41%	0.28%	0.23%	0.21%	0.16%	0.16%	0.10%	0.16%	0.16%	0.14%	0.10%	0.10%	0.08%	0.08%	0.06%	0.00%	0.00%	0.00%	0.00%

### Differential expression analyses

**Figure 1:**
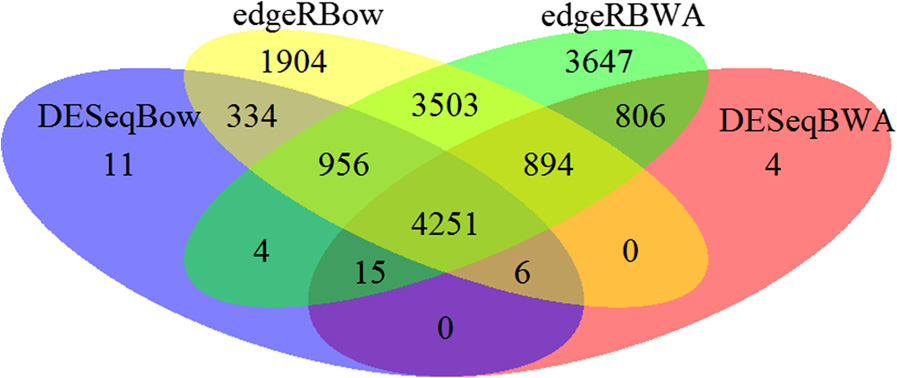
**Overlap between the number of differentially expressed transfrags detected from the four combinations of mapping and significance testing methods for sequences involved in transcriptomic response to increased temperature for the rainbowfish *Melanotaenia duboulayi*.** See text for details of mapping and testing methods used.

**Figure 2:**
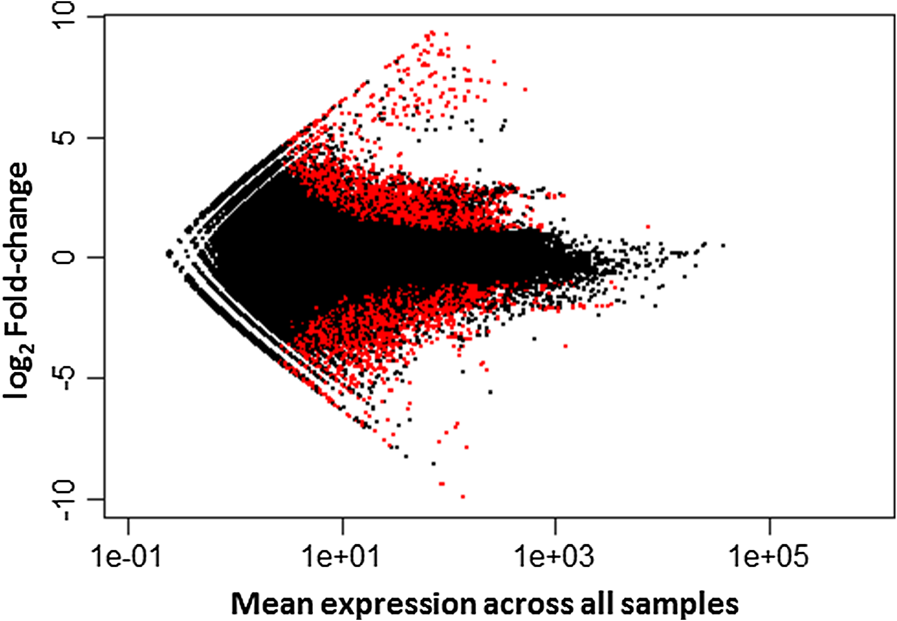
**Differential expression of 107,749 transfrags assembled for the rainbowfish *Melanotaenia duboulayi *under different temperature treatments (21°C vs. 33°C).** Results are shown as the log_2_ fold change in expression versus the mean expression level between treatment groups. Red dots above zero fold change represent significantly up-regulated transfrags whereas red dots below zero fold change represent significantly down-regulated tranfrags at the 0.5 false discovery rate.

The four different combinations of mapping and DE testing produced vastly different numbers of DE transfrags (see Table 1, Figure 1). The combination of BWA alignment followed by EdgeR DE analysis identified the most with 14,076 DE transfrags, whereas Bowtie followed by DESeq identified the least with 5,577 (Figure 1). The difference between the approaches likely arises from the different characteristics of the two aligners combined with the sensitivities of the DE tests. Bowtie does not allow gapped alignments and makes use of the base quality scores [49], making it more conservative than BWA in the number of mapped reads. On the other hand DESeq has also been shown to be more conservative than EdgeR when identifying DE genes from low count data [50] which likely explains the lower number of hits in multiplex sequencing strategies such as ours. The total number of DE transfrags identified by all four approaches was 4,251 (Figure 2). We adopted a conservative approach and selected only these transfrags to blast against the reference database. Future RNA-seq studies should assess their priorities for DE gene discovery and select the detection strategy based upon the need for identifying lowly expressed genes versus the accuracy expected given the number of replicates used [51]. Robles *et al.*[50] showed that EdgeR could be used to detect higher numbers of DE transfrags from low count data without compromising accuracy when the number of biological replicates was at least six in each treatment group.

The Blast2GO program was able to find sequence similarities for 2,740 of the DE transfrags but could not find mapping or annotation information for a further 634 of them, leaving 2,106 DE transfrags which were successfully annotated. The top 15 matching species from the BLAST query were all fish species with the most BLAST hits being for the Nile tilapia *Oreochromis niloticus* with 583 matches. Duplicate gene isoforms were detected by matching identical annotated gene names from the Blast2GO output. These isoforms were then combined and reported as single “genes”. Once isoforms were combined, there were 614 genes that were up-regulated in the high temperature treatment with 349 genes being down-regulated (see Additinal file 1: Table S2a and b). For significantly down-regulated transfrags, the mean fold-change between ambient and high-temperature conditions was 4.0-fold, with a range from 55.6-fold for g2/m phase specific e3 ubiquitin-protein ligase to 2.2-fold for the Phytanoyl-peroxisomal-like protein. The mean fold-change for significantly up-regulated transfrags was 11.13, ranging from 1.98 (for the cyclin-dependent kinase 2 interacting protein) to 259-fold (for the Heat shock protein Hsp-90-like).

### Ontology of differentially expressed genes

**Figure 3:**
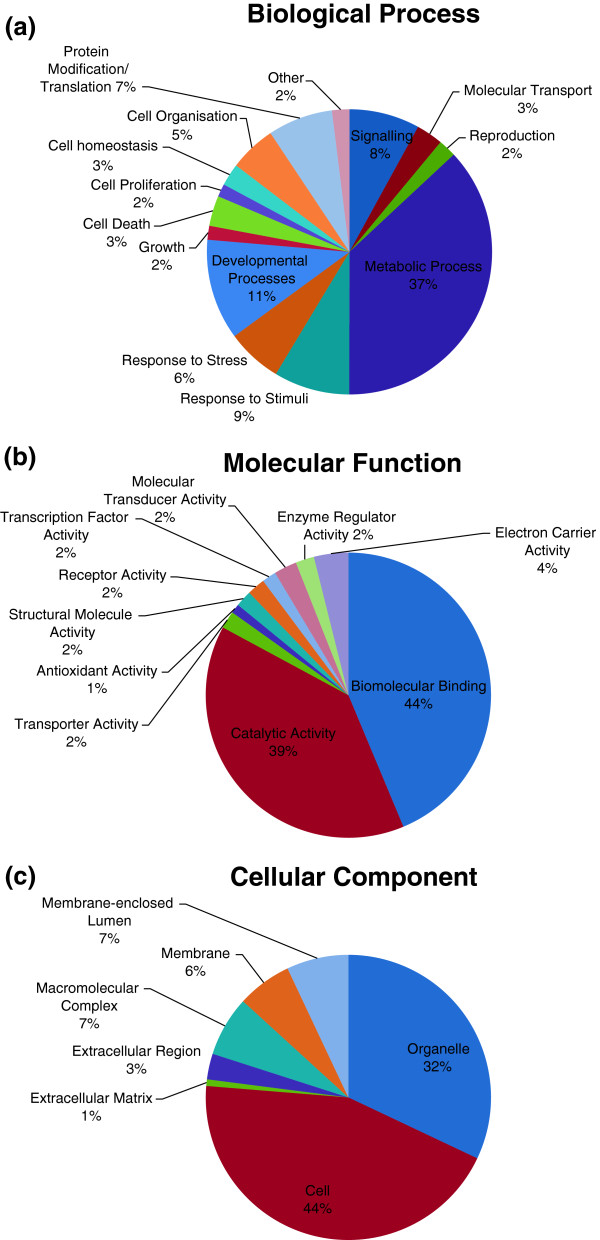
**Distribution of annotated transfrags assigned to (a) biological processes or (b) molecular functions or (c) the cellular components according to gene ontology association.** Analysis carried out with the Blast2Go program for sequences involved in transcriptomic response to increased temperature for the rainbowfish *Melanotaenia duboulayi*.

Many functional classes of genes were affected by temperature stress. As expected, heat shock protein genes including *HSPA4* (12.3 x), *Hsp60* (6.6 x), *Hsp70* (9.9 x) and *Hsp90α* (141.3 x) were significantly up-regulated in heat stressed fish. These transcripts are well characterised as stress inducible and have been shown, in many species, to be involved in protection against apoptosis or as a molecular chaperone under extended exposure to heat stress [15,19,20,52-56]. Further to these well-characterised stress related genes, the gene ontology analysis also identified transcripts involved in catabolism (11% of annotated sequences) and lipid metabolism (12% of annotated sequences) as being the important biological processes in the response to temperature stress (Figure 3a). As with other studies in fish, regulation of metabolic processes are clearly important parts of the heat stress response [21,22,24]. A large proportion of the individual over-expressed genes in rainbowfish were related to oxidoreductase activity, mitochondrial components and organelle membranes. These gene categories are typically associated with increased metabolism, particularly to cope with increased temperature and the related hypoxic conditions. Additionally we found a role for genes of the ubiquitin family and the gene 78 kDa glucose-regulated protein precursor which, similar to Quinn *et al.*[57], were upregulated in response to heat stress. Gene ontology analysis also identified biomolecular binding and catalytic activity as the major molecular functions affected by exposure to different temperature regimes (see Figure 3b). Within these broad categories, protein binding and ATP binding were the major biomolecular binding functions affected by differentially expressed transfrags with node scores of 244 and 226 respectively. For catalytic activity, transferase activity (nodescore = 53) and oxidoreductase activity were prominent (node score = 54). These functional categories, combined with electron carrier activity (node score = 63), is congruent with the expected role of aerobic respiration in response to the increased temperature.

While the *Hsp* genes are commonly identified as overexpressed in short-term temperature manipulation experiments [24,37], they are less likely to be targets for selection during gradual temperature shifts associated with climate change [22,53]. *Hsp* genes represent a physiological response to sudden stressors and therefore plasticity in these traits is unlikely to be adaptive over longer timescales [58]. The more likely candidates for an adaptive genetic response are those genes involved in what has been termed the “cellular homeostatis response” to long-term temperature stress [59]. Unlike stress response genes that provide an immediate early response to macromolecular damage and sudden changes in cellular redox potential, the cellular homeostatasis response involves effector proteins mediating parameter specific adaptation to environmental change.

### Responses associated with prolonged exposure to heat stress

Prolonged exposure to increased temperatures has previously been associated with gene ontologies related to protein folding, oxidative stress and immune function [18,19]. Similarly, we detected significant upregulation of genes with these ontologies in the high temperature treatment such as Calnexin (2.8 x), NADH dehydrogenase (2.5 x), and glutathione S-transferase (5.1 x) suggesting long-term reallocation of energy resources. Plasticity in the expression of these genes is more likely to be adaptive and allow localised populations to survive in a changing environment, eventually leading to divergent selection. Kassahn *et al.*[53] grouped stress-response transcripts into four different clusters according to the pattern of regulation detected under short versus long-term exposure to heat stress. They suggested that long-term exposure to heat stress in a coral reef fish (31°C for five days) induces expression of genes involved in development and immune function whereas genes related to metabolic function are suppressed. Our data, from long-term exposure to heat stress in rainbowfish (33°C for 14 days), support those findings. Developmental processes and metabolic processes accounted for 48% of dysregulated transfrags (Figure 3a). Immune function seems less important in our dataset and is covered by the “response to stimuli” category representing 9% of DE transfrags including the natural killer cell enhancement factor (upregulated 2.8 x). It is possible that the longer exposure to heat stress in our study allowed recovery from the immediate activation of the immune function genes.

Under simulated models of divergence with plasticity, selection is possible when plasticity is moderate, dispersal ability is low and there are no fitness costs to plasticity [60]. It may therefore be worthwhile to focus attention on those gene regions that showed mid-range shifts in expression level in the treatment group when looking for evidence of adaptive evolution. In particular, the mid-range transfrags related to metabolic and developmental processes as well as immune function are likely to be good candidates as genes of adaptive significance for increasing temperatures (Table 2). Rainbowfish represent ideal candidates for studies of local adaptation due to their reduced dispersal and distribution over multiple ecoregions. The suite of genes that we present here showing a response to increased temperature are a good starting point for further manipulative experiments or landscape wide surveys of genetic variation. Creating a catalogue of polymorphisms at these genes throughout the range of *M. duboulayi* will provide insights into the adaptive potential of this species in the face of a warming environment.

**Table 2:** **Candidate genes for broad scale studies of temperature response in the crimson spotted rainbowfish, *Melanotaenia duboulayi***

**Seq. description**	**min. e value**	**Fold change**	**Primary gene Ontology**
3-keto-steroid reductase-like	5.09E-49	2.25	C:endoplasmic reticulum membrane
hydroxymethylglutaryl- cytoplasmic	9.30E-32	2.25	P:isoprenoid biosynthetic process
smooth muscle cell-specific protein sm22 alpha	1.44E-126	2.28	P:muscle organ development
phospholemman precursor	1.98E-44	2.32	C:membrane
ap-2 complex subunit sigma	1.34E-91	2.32	P:axon guidance
acyl carrier mitochondrial precursor	1.87E-82	2.34	F:phosphopantetheine binding
nadh-cytochrome b5 reductase 2	3.52E-137	2.34	F:cytochrome-b5 reductase activity
sterol-4-alpha-carboxylate 3- decarboxylating-like	7.81E-44	2.36	P:steroid biosynthetic process
retinol-binding protein cellular	3.29E-86	2.38	P:transport
ribosomal rna processing protein 36 homolog	9.82E-58	2.38	C:nucleolus
protein cdv3 homolog	4.64E-64	2.38	C:cytoplasm
nadh dehydrogenase 1 alpha subcomplex subunit 6	1.63E-77	2.41	C:mitochondrial inner membrane
y chain e2~ubiquitin-hect	1.04E-45	2.41	P:endosome transport
monoamine oxidase	0	2.42	P:catecholamine metabolic process
small nuclear ribonucleoprotein sm d2	2.44E-64	2.42	P:ncRNA metabolic process
transketolase	6.63E-137	2.42	F:transketolase activity
rho-class glutathione s-transferase	1.95E-101	2.43	F:transferase activity
thioredoxin domain-containing protein 14 precursor	2.31E-72	2.44	P:cell redox homeostasis
ubiquitin-conjugating enzyme e2 variant 2	1.43E-85	2.45	F:acid-amino acid ligase activity
coiled-coil domain-containing protein 47 precursor	0	2.46	P:embryonic development
ubiquinol-cytochrome c reductase core protein ii	0	2.47	F:metalloendopeptidase activity
3-hydroxy-3-methylglutaryl-coenzyme a synthase 1	8.22E-40	2.99	P:response to tellurium ion
nuclear factor erythroid derived 2-like 1	2.00E-60	3.24	P:heme biosynthetic process
glutathione s-transferase	3.32E-30	5.07	F:glutathione transferase activity
cyclin-dependent kinase inhibitor 1	6.69E-65	5.86	P:cellular response to stimulus
catechol-o-methyltransferase domain-containing protein 1	1.45E-75	−2.44	F:O-methyltransferase activity
period homolog 3	1.01E-173	−2.44	C:cytoplasm
histamine n-methyltransferase	1.19E-130	−2.50	P:respiratory gaseous exchange
scinderin like a	0	−2.50	P:eye development
5-aminolevulinate erythroid- mitochondrial-like	0	−2.50	P:response to hypoxia
55 kda erythrocyte membrane protein	2.00E-91	−2.50	C:intracellular non-membrane-bounded organelle
plakophilin 3	0	−2.50	F:binding
cbp p300-interacting transactivator 3b	5.19E-44	−2.50	C:nucleus
lysosomal alpha-glucosidase-like	0	−2.56	F:carbohydrate binding
actin-binding lim protein 1 long isoform isoform cra_a	4.22E-35	−2.56	P:axon guidance
udp-glucuronosyltransferase 2a2-like isoform 2	1.10E-93	−2.56	F:transferase activity, transferring hexosyl groups
glucose-fructose oxidoreductase domain-containing protein 1-like	4.30E-38	−2.56	C:extracellular region
dual specificity tyrosine-phosphorylation-regulated kinase 1b	0	−2.56	P:protein amino acid autophosphorylation
synaptobrevin homolog ykt6	3.21E-121	−2.56	C:Golgi membrane
serine--pyruvate mitochondrial precursor	2.50E-25	−2.56	P:metabolic process
transmembrane protein 192	6.58E-112	−2.63	C:membrane
protein creg2-like	9.34E-142	−2.63	C:cytoplasmic part
ras-related protein rab-13-like	1.31E-67	−2.63	P:vesicle-mediated transport
c-jun-amino-terminal kinase-interacting protein 4 isoform partial	0	−2.63	F:protein binding
histone-lysine n-methyltransferase setd3-like	3.00E-148	−2.63	P:peptidyl-lysine monomethylation
peroxisome proliferator-activated receptor alpha	2.04E-78	−2.86	P:steroid hormone mediated signaling pathway
cytochrome p450 1a	5.02E-152	−3.70	C:endoplasmic reticulum membrane
thyrotrophic embryonic factor	9.16E-157	−3.85	P:cellular response to light stimulus
nuclear receptor subfamily 1 group d member 2	3.54E-102	−4.17	P:steroid hormone mediated signaling pathway
vitellogenin ab	0	−10.0	F:lipid transporter activity

Genes correspond to transfrags with mid-range differential expression values that match metabolic, developmental, or immune response processes from gene ontology assignment by the Blast2Go program. Gene ontology abbreviations: P= biological process, F= molecular function, C= cellular component.

### RNA-seq recommendations for non-model taxa

The results of this study highlight the appropriateness of an RNA-seq approach for studies of adaptation (including adaptive plasticity) in non-model organisms. With the paucity of genomic resources available for most wildlife species, NGS technologies offer the best hope for unravelling the processes of evolutionary adaptation in a natural setting. Rainbowfish are evolutionarily very different from their nearest genome-enabled species, *Oryzias latipes*, yet in this study we were able to generate a substantial list of candidate genes involved in a response to increasing temperatures. Over the past few years, the proliferation of software resources and validated pipelines for RNA-seq means that virtually any organism can now be the focus of ecological genomic research and this is reflected in the rapid increase in publications reporting RNA-seq analyses in non-human taxa. The limiting factors that remain now are bioinformatic expertise and incomplete reference data. Over half of the dysregulated transfrags identified in our study were unable to be identified or were of unknown function. This continues to be a major challenge for studies of ecological and evolutionary genomics [6]. Interpretation of genomic data lags well behind the current ability to generate that data. The limitation stems from the fact that annotation of genes of ecological interest still relies upon inferring homologies with genomic features established and developed in a few model species for non-ecological purposes. Better data integration is needed to facilitate the association of gene transcripts with specific natural conditions or phenotypic responses. Further work to characterise the function of these unknown genes via experimental studies of non-model organisms will enhance our understanding of the important biological pathways involved in responses to temperature stress and other environmental changes. We have shown that differing mapping and DE analysis approaches lead to very different outcomes in terms of the DE genes identified. While a combination of all available approaches is preferable to identify overlap in the candidate genes detected, we found that combining output from just Bowtie mapping and DESeq significance testing with BWA mapping and DESeq significance testing delivered just 21 more DE genes than combining all four approaches tested in our study (see Figure 1). This conservative approach is an efficient way to avoid large numbers of false positives being detected in RNA-seq studies.

## Conclusions

Temperature increases predicted over the coming decades suggests species with limited dispersal abilities will need substantial adaptive potential to avoid extinction. That adaptive potential will likely come from a number of sources including adaptive phenotypic plasticity, standing genetic variation, and newly-derived mutations. Regardless of the source, adaptation will be most important in those processes related to heat tolerance. We have presented a first insight into which processes are likely to be important in the rainbowfish, *M. duboulayi*. This provides a foundation for future research into temperature-driven adaptive responses in freshwater species but also invites more detailed study of the phenome-genome interaction under conditions of temperature stress.

We identified a predictable suite of heat shock genes that responded sharply to increased temperatures in the treatment group. However, we also identified transfrags related to regulation of metabolic functions and developmental processes that showed mid-range levels of dysregulation and may be stronger candidates as genes for long-term adaptation to a warming environment. We present these candidate genes as targets for ongoing research into populations representing different thermal environments throughout the species range. We also expect that these candidates will be useful targets for studies of other freshwater species experiencing long-term thermal challenges.

The expression level changes we have presented may be an example of a plastic response. To check for an adaptive component it is necessary to repeat the temperature trial on other geographically distant populations and/or sister taxa. Parallel expression level changes in these populations would indicate plasticity whereas altered responses would be suggestive of adaptation at the genome level. Such “common garden” experiments allow the disentangling of pure plastic vs. genetic responses and are ideal approaches for future research. Other avenues to explore evolutionary adaptation to increased temperatures include investigating if DNA polymorphisms are present within and between populations at the gene regions we have identified in this study. Extensions of this research to include adaptive traits from other important environmental impacts will enable a much broader understanding of how freshwater species are likely to cope with human-induced habitat and climatic change.

## Availability of supporting data

Raw sequencing data is available through the NCBI Sequence Read Archive under Project ID PRJNA205235 (http://trace.ncbi.nlm.nih.gov/Traces/sra/). All samples were sequenced as 101 bp paired-end reads on an Illumina HiSeq2000 sequencer.

## Competing interests

The authors declare that they have no competing interests.

## Authors' contributions

SS participated in the study planning and coordination, carried out the molecular genetic component, analysed the genomic data and drafted the manuscript. LBB conceived the study, participated in its design and coordination and helped to draft the manuscript. LB participated in the study design and planning and helped to draft the manuscript. All authors read and approved the final manuscript.

## Supplementary Material

Additional file 1: Table S1 Sequencing statistics for individual paired end reads from the pooled RNA-Seq library from *M. duboulayi* sequenced in a single lane of the Illumina HiSeq 2000. **Table S2a**. Annotated genes matching up-regulated transfrags in the high temperature group of *M. duboulayi*. Mean similarity is computed as the average similarity value for all the hits of a given sequence. Gene ontology abbreviations: P= biological process, F= molecular function, C= cellular component. **Table S2b**. Annotated genes matching down-regulated transfrags in the high temperature group of *M. duboulayi*. Mean similarity is computed as the average similarity value for all the hits of a given sequence. Click here for file
