# Supplementary material for: RNA-seq analysis reveals extensive transcriptional plasticity to temperature stress in a freshwater fish species
Source: BMC Genomics. 2013 Jun 5;14:375. doi: 10.1186/1471-2164-14-375 (PMC3680095; doi:10.1186/1471-2164-14-375)
Supplement: Additional file 1: Table S1 — Sequencing statistics for individual paired end reads from the pooled RNA-Seq library from M. duboulayi sequenced in a single lane of the Illumina HiSeq 2000. Table S2a. Annotated genes matching up-regulated transfrags in the high temperature group of M. duboulayi. Mean similarity is computed as the average similarity value for all the hits of a given sequence. Gene ontology abbreviations: P= biological process, F= molecular function, C= cellular component. Table S2b. Annotated genes matching down-regulated transfrags in the high temperature group of M. duboulayi. Mean similarity is computed as the average similarity value for all the hits of a given sequence. [file 1471-2164-14-375-S1.docx]

**Supplementary Tables**

**Supplementary Table 1:** Sequencing statistics for individual paired end reads from the pooled RNA-Seq library from *M. duboulayi* sequenced in a single lane of the Illumina HiSeq 2000.

| Sample Name | | Raw Reads | | Filtered Reads |
| --- | --- | --- | --- | --- |
| Cold Group | Md21_1b | | 16349130 | 14574082 |
|  | Md21_5a | | 14432518 | 11696942 |
|  | Md21_6b | | 28693838 | 25401102 |
|  | Md21_8b | | 19641630 | 17847442 |
|  | Md21_9b | | 15586224 | 13838678 |
|  | Md21_10a | | 32182792 | 28983334 |
|  | **Total Cold** | | **126886132** | **112341580** |
| Warm Group | Md35_2a | | 21572230 | 18934578 |
|  | Md35_3b | | 20234400 | 17870568 |
|  | Md35_6b | | 22913948 | 19990770 |
|  | Md35_7b | | 16577394 | 13940082 |
|  | Md35_9a | | 24434198 | 21509642 |
|  | Md35_10b | | 23292216 | 19803098 |
|  | **Total Warm** | | **129024386** | **112048738** |
| Total Reads |  | | **255910518** | **224390318** |

**Supplementary Table 2a**: Annotated genes matching **up**-regulated transfrags in the high temperature group of *M. duboulayi*. Mean similarity is computed as the average similarity value for all the hits of a given sequence. Gene ontology abbreviations: P= biological process, F= molecular function, C= cellular component.

| **Seq. Description** | **Seq. Length** | **min. eValue** | **mean Similarity** | **Primary Gene Ontology** |
| --- | --- | --- | --- | --- |
| **acetyl-coenzyme a cytoplasmic** | 602 | 4.63E-105 | 81.60% | C:nucleolus |
| **isopentenyl-diphosphate delta-isomerase 1** | 434 | 7.81E-34 | 86.30% | F:metal ion binding |
| **3-hydroxy-3-methylglutaryl-coenzyme a reductase** | 493 | 6.35E-98 | 89.90% | P:coenzyme A metabolic process |
| **hmg- reductase** | 435 | 3.53E-60 | 96.80% | P:coenzyme A metabolic process |
| **3-beta-hydroxysteroid-delta -isomerase-like** | 300 | 4.38E-42 | 84.50% | F:cholestenol delta-isomerase activity |
| **3-beta-hydroxysteroid-delta -isomerase** | 216 | 4.16E-42 | 89.60% | P:hemopoiesis |
| **acetyl- carboxylase alpha** | 3239 | 0 | 96.50% | P:long-chain fatty-acyl-CoA biosynthetic process |
| **ap-2 complex subunit sigma** | 1826 | 1.34E-91 | 98.50% | P:axon guidance |
| **nadh-cytochrome b5 reductase 2** | 920 | 3.52E-137 | 86.00% | F:cytochrome-b5 reductase activity |
| **a chain thermodynamic and structure guided design of statin hmg-coa reductase inhibitors** | 304 | 2.32E-52 | 92.30% | P:coenzyme A metabolic process |
| **star-related lipid transfer protein 5-like** | 1317 | 2.62E-94 | 74.90% | P:cholesterol transport |
| **cytochrome family subfamily polypeptide 1** | 1611 | 9.00E-30 | 97.80% | P:oxidation reduction |
| **lanosterol 14-alpha demethylase-like** | 446 | 4.54E-47 | 90.10% | C:endoplasmic reticulum membrane |
| **14-alpha demethylase** | 296 | 9.88E-43 | 93.10% | C:endoplasmic reticulum membrane |
| **ubiquitin c** | 357 | 8.49E-77 | 100.00% | P:viral reproduction |
| **d chain crystal structure of the mouse hoil1-l-nzf in complex with linear di- ubiquitin** | 412 | 2.63E-43 | 100.00% | P:activation of MAPK activity involved in innate immune response |
| **achain crystal structure of a linear-specific ubiquitin fab bound to linear ubiquitin** | 326 | 7.12E-51 | 99.80% | P:viral reproduction |
| **ubiquitin c variant 4** | 450 | 1.78E-98 | 100.00% | C:nucleus |
| **y chain e2~ubiquitin-hect** | 371 | 1.04E-45 | 99.70% | P:endosome transport |
| **seryl-trna synthetase** | 2011 | 0 | 93.60% | C:cytosol |
| **acetoacetyl- synthetase** | 419 | 3.65E-86 | 95.40% | P:cellular response to testosterone stimulus |
| **acetoacetyl- synthetase-like** | 411 | 3.72E-36 | 92.70% | C:cytosol |
| **hydroxymethylglutaryl- cytoplasmic** | 711 | 9.30E-32 | 80.80% | P:isoprenoid biosynthetic process |
| **hydroxymethylglutaryl- cytoplasmic-like** | 704 | 7.24E-96 | 87.90% | C:cytoplasm |
| **3-hydroxy-3-methylglutaryl-coenzyme a synthase 1** | 212 | 8.22E-40 | 95.50% | P:response to tellurium ion |
| **acetyl- carboxylase** | 331 | 2.45E-39 | 98.60% | C:cytoplasm |
| **acetyl- cytosolic** | 605 | 6.96E-122 | 89.10% | C:nucleolus |
| **alpha-1-antitrypsin homolog** | 825 | 2.59E-111 | 75.00% | F:peptidase inhibitor activity |
| **alpha- partial** | 188 | 7.09E-38 | 99.80% | C:cytosol |
| **tubulin alpha** | 293 | 5.61E-51 | 99.60% | C:microtubule |
| **alpha-tubulin i** | 404 | 1.15E-79 | 99.80% | C:microtubule |
| **tubulin alpha-1c chain- partial** | 390 | 6.70E-43 | 100.00% | P:'de novo' posttranslational protein folding |
| **tubulin alpha-1a chain isoform 2** | 1309 | 0 | 99.00% | P:'de novo' posttranslational protein folding |
| **7-alpha-hydroxycholest-4-en-3-one 12-alpha-hydroxylase-like** | 2173 | 0 | 83.00% | F:heme binding |
| **glutathione s-transferase** | 424 | 3.32E-30 | 83.00% | F:glutathione transferase activity |
| **rho-class glutathione s-transferase** | 847 | 1.95E-101 | 88.80% | F:transferase activity |
| **lanosterol 14alpha-demethylase** | 151 | 6.18E-21 | 91.50% | F:sterol 14-demethylase activity |
| **calumenin precursor** | 771 | 2.70E-82 | 79.00% | C:sarcoplasmic reticulum |
| **eukaryotic initiation factor 4a-ii-like** | 289 | 1.53E-58 | 100.00% | F:translation initiation factor activity |
| **eukaryotic translation initiation factor isoform 2** | 375 | 2.48E-76 | 94.70% | F:translation initiation factor activity |
| **eukaryotic translation initiation factor isoform isoform cra_b** | 452 | 1.30E-78 | 100.00% | F:translation initiation factor activity |
| **eukaryotic initiation factor 4a-ii** | 1840 | 0 | 97.40% | F:translation initiation factor activity |
| **heat shock protein 90 alpha** | 599 | 1.73E-114 | 97.40% | P:axon guidance |
| **heat shock protein hsp 90-alpha-like** | 1135 | 0 | 94.40% | P:protein folding |
| **udp- partial** | 204 | 7.04E-26 | 85.90% | F:transferase activity, transferring hexosyl groups |
| **udp glucuronosyltransferase 1 family polypetide partial** | 364 | 3.95E-64 | 87.20% | F:transferase activity, transferring hexosyl groups |
| **ugt1ab protein** | 619 | 4.89E-36 | 90.30% | F:hydrolase activity |
| **udp-glucuronosyltransferase** | 1421 | 0 | 77.70% | F:transferase activity, transferring hexosyl groups |
| **calr protein** | 1083 | 0 | 93.10% | P:protein folding |
| **calreticulin-like isoform 1** | 1291 | 0 | 92.90% | P:protein folding |
| **calreticulin precursor** | 446 | 3.17E-45 | 94.40% | P:protein folding |
| **transferrin receptor protein 2-like** | 401 | 1.95E-73 | 78.30% | F:receptor activity |
| **protein disulfide-isomerase a4** | 1381 | 0 | 90.50% | C:endoplasmic reticulum lumen |
| **alanine aminotransferase 2-like** | 978 | 1.34E-20 | 93.20% | F:pyridoxal phosphate binding |
| **transketolase** | 1323 | 6.63E-137 | 90.10% | F:transketolase activity |
| **tkt protein** | 1408 | 4.60E-106 | 84.50% | F:metal ion binding |
| **transketolase-like protein 2** | 348 | 6.67E-54 | 90.70% | P:regulation of growth |
| **transmembrane emp24 domain-containing protein 10 precursor** | 1683 | 1.47E-127 | 95.50% | P:transport |
| **tyrosyl-trna synthetase** | 817 | 5.05E-161 | 97.50% | C:cytosol |
| **pyrroline-5-carboxylate reductase 2** | 709 | 3.83E-159 | 95.60% | F:binding |
| **ddb1- and cul4-associated factor 13** | 1249 | 0 | 92.00% | C:nucleolus |
| **3-keto-steroid reductase-like** | 278 | 5.09E-49 | 85.80% | C:endoplasmic reticulum membrane |
| **hydroxysteroid (17-beta) dehydrogenase 7** | 355 | 2.17E-34 | 82.80% | C:endoplasmic reticulum membrane |
| **6-phosphogluconate decarboxylating** | 349 | 6.28E-47 | 92.70% | F:NADP or NADPH binding |
| **6-phosphogluconate dehydrogenase** | 149 | 3.79E-26 | 93.20% | C:cytoplasm |
| **elongation of very long chain fatty acids protein 6** | 716 | 4.42E-95 | 95.60% | F:transferase activity, transferring acyl groups other than amino-acyl groups |
| **tumor rejection antigen 1** | 983 | 0 | 97.40% | P:ER-associated protein catabolic process |
| **glucose-regulated protein 94** | 582 | 1.24E-48 | 97.90% | P:ER-associated protein catabolic process |
| **achain grp94 n-terminal domain bound to geldanamycin: effects of mutants 168- 169 ks-aa** | 288 | 4.68E-20 | 100.00% | P:ER-associated protein catabolic process |
| **endoplasmin precursor** | 483 | 6.65E-39 | 94.40% | P:response to hypoxia |
| **squalene epoxidase** | 297 | 1.66E-35 | 92.60% | C:endoplasmic reticulum membrane |
| **u3 small nucleolar ribonucleoprotein protein imp3** | 724 | 5.99E-118 | 93.70% | F:rRNA binding |
| **glucosamine--fructose-6-phosphate aminotransferase** | 3147 | 0 | 95.30% | P:negative regulation of glycogen biosynthetic process |
| **aldehyde dehydrogenase family 1 member l1-like** | 427 | 1.67E-87 | 94.20% | F:acyl carrier activity |
| **78 kda glucose-regulated protein precursor** | 1086 | 6.19E-141 | 96.50% | F:ATP binding |
| **carbamoyl-phosphate synthetase aspartate and dihydroorotase** | 1163 | 0 | 95.50% | P:response to amine stimulus |
| **heat shock protein 60 kda** | 230 | 2.05E-40 | 95.80% | P:protein refolding |
| **retinol-binding protein cellular** | 868 | 3.29E-86 | 88.80% | P:transport |
| **asparaginyl-trna cytoplasmic** | 1342 | 1.12E-142 | 96.80% | F:nucleic acid binding |
| **lanosterol synthase** | 946 | 4.82E-145 | 91.00% | F:intramolecular transferase activity |
| **calnexin precursor** | 616 | 5.99E-31 | 88.40% | C:endoplasmic reticulum membrane |
| **udp-glucose 6-dehydrogenase isoform 2** | 821 | 2.77E-164 | 95.30% | P:gastrulation with mouth forming second |
| **udp-glucose 6-dehydrogenase** | 923 | 8.50E-48 | 83.90% | F:oxidoreductase activity, acting on the CH-OH group of donors, NAD or NADP as acceptor |
| **pre-mrna-splicing factor atp-dependent rna helicase dhx15-like** | 1722 | 0 | 95.90% | F:ATP-dependent helicase activity |
| **dead (asp-glu-ala-asp) box polypeptide 56** | 471 | 4.57E-85 | 87.00% | F:RNA binding |
| **coiled-coil domain-containing protein 47 precursor** | 2035 | 0 | 92.50% | P:embryonic development |
| **lathosterol oxidase** | 1522 | 1.95E-158 | 87.80% | P:fatty acid biosynthetic process |
| **atp-binding cassette sub-family f member 1** | 692 | 1.18E-22 | 72.30% | F:nucleoside-triphosphatase activity |
| **atp-binding sub-family f member 1** | 667 | 1.67E-27 | 66.90% | P:translational initiation |
| **stress-induced-phosphoprotein 1** | 1187 | 0 | 95.60% | P:response to stress |
| **succinate dehydrogenase** | 1293 | 0 | 95.20% | P:transport |
| **autosomal recessive 1a** | 394 | 7.43E-63 | 77.20% | C:nucleolus |
| **lysophospholipid acyltransferase 7** | 2176 | 8.26E-154 | 79.90% | F:acyltransferase activity |
| **protein tyrosine phosphatase-like a domain containing 1** | 1504 | 2.37E-180 | 86.50% | C:endoplasmic reticulum membrane |
| **novel protein (zgc:56258)** | 420 | 1.62E-26 | 78.20% | C:nucleolus |
| **trna guanosine-2 -o-methyltransferase trm13 homolog** | 415 | 2.34E-46 | 79.10% | F:methyltransferase activity |
| **glutathione synthetase** | 344 | 3.68E-56 | 83.60% | P:response to cadmium ion |
| **neutral alpha-glucosidase ab-like** | 517 | 6.35E-94 | 88.40% | F:carbohydrate binding |
| **lon protease mitochondrial** | 992 | 2.15E-160 | 88.00% | P:cellular chaperone-mediated protein complex assembly |
| **t-complex protein 1 subunit epsilon** | 477 | 2.89E-58 | 99.50% | C:nucleolus |
| **dead (asp-glu-ala-asp) box polypeptide 3** | 1117 | 0 | 89.50% | C:cytoplasm |
| **phosphatidylinositol-4-phosphate 3-kinase c2 domain-containing subunit gamma-like** | 2070 | 0 | 72.20% | P:cellular process |
| **uridine 5 -monophosphate synthase** | 1618 | 0 | 81.10% | P:'de novo' UMP biosynthetic process |
| **nhp2-like protein 1** | 687 | 1.33E-69 | 99.40% | C:nucleolus |
| **succinyl- ligase** | 658 | 1.66E-129 | 96.30% | F:metal ion binding |
| **toll interleukin-1 receptor domain-containing adapter protein** | 599 | 2.35E-76 | 64.70% | F:receptor activity |
| **asparagine synthetase** | 805 | 3.36E-145 | 84.10% | F:asparagine synthase (glutamine-hydrolyzing) activity |
| **transmembrane 7 superfamily member 2** | 368 | 1.47E-49 | 69.00% | P:lipid biosynthetic process |
| **delta -sterol reductase** | 618 | 1.09E-84 | 72.30% | C:membrane |
| **delta -sterol reductase-like** | 1136 | 2.72E-171 | 87.60% | C:endoplasmic reticulum membrane |
| **glutathione reductase** | 304 | 1.34E-54 | 89.70% | C:cytoplasm |
| **sterol-4-alpha-carboxylate 3- decarboxylating-like** | 947 | 7.81E-44 | 89.60% | P:steroid biosynthetic process |
| **ribosome biogenesis protein bms1 homolog** | 1005 | 1.71E-179 | 88.00% | P:GTP catabolic process |
| **myb-binding protein 1a-like** | 1566 | 0 | 73.20% | C:nucleolus |
| **glutamate dehydrogenase 1** | 1441 | 0 | 96.10% | F:glutamate dehydrogenase [NAD(P)+] activity |
| **retinol dehydrogenase 12** | 548 | 3.45E-90 | 81.20% | C:endoplasmic reticulum membrane |
| **probable dimethyladenosine transferase** | 991 | 9.03E-162 | 96.40% | C:cytoplasm |
| **thioredoxin reductase 1** | 972 | 0 | 92.80% | P:cell redox homeostasis |
| **prostaglandin e synthase 2-like** | 895 | 0 | 87.10% | P:prostaglandin biosynthetic process |
| **mitochondrial 39s ribosomal protein l33** | 508 | 1.46E-34 | 92.40% | C:ribosome |
| **protein disulfide-isomerase a5-like** | 1068 | 0 | 91.70% | P:cell redox homeostasis |
| **protein fam136a-like** | 943 | 8.23E-83 | 90.40% | C:mitochondrion |
| **transmembrane emp24 domain-containing protein 9 precursor** | 820 | 1.20E-130 | 98.20% | P:transport |
| **dead box rna helicase-pl10a** | 294 | 6.50E-57 | 97.60% | C:cytoplasm |
| **heterogeneous nuclear ribonucleoprotein h1** | 591 | 9.63E-119 | 94.60% | C:cytoplasm |
| **nucleolar protein 14** | 479 | 1.50E-76 | 80.20% | C:mitochondrion |
| **clarin 1** | 787 | 1.16E-67 | 76.40% | P:sensory perception of sound |
| **leucine-rich repeat-containing protein 47** | 1410 | 0 | 80.80% | F:RNA binding |
| **peroxisomal lon protease homolog 2** | 1283 | 8.47E-28 | 88.20% | P:signal peptide processing |
| **transmembrane protein 33** | 632 | 2.17E-130 | 90.70% | C:membrane |
| **glutathione s-transferase omega-1-like** | 533 | 1.67E-88 | 90.50% | P:metabolic process |
| **cytosol aminopeptidase** | 1340 | 0 | 82.50% | F:metalloexopeptidase activity |
| **lon protease homolog peroxisomal-like** | 1121 | 0 | 88.00% | P:signal peptide processing |
| **lon protease homolog peroxisomal** | 727 | 1.84E-131 | 85.00% | P:signal peptide processing |
| **lon peptidase peroxisomal** | 1801 | 0 | 86.10% | P:signal peptide processing |
| **npm1 protein** | 664 | 1.51E-27 | 67.00% | F:protein binding |
| **nucleophosmin** | 2223 | 3.55E-72 | 86.20% | C:nucleolus |
| **glucose-6-phosphate 1-dehydrogenase** | 1629 | 1.95E-162 | 94.00% | P:glucose metabolic process |
| **cysteine and histidine-rich domain-containing protein 1** | 887 | 1.17E-166 | 86.70% |  |
| **atp-binding cassette sub-family e member 1** | 1562 | 0 | 89.90% | F:ATPase activity |
| **ubiquinol-cytochrome c reductase core protein ii** | 1629 | 0 | 86.30% | F:metalloendopeptidase activity |
| **programmed cell death protein 4** | 550 | 2.98E-89 | 87.90% | F:binding |
| **ubiquitin-conjugating enzyme e2 variant 2** | 1007 | 1.43E-85 | 94.90% | F:acid-amino acid ligase activity |
| **solute carrier family 12 member 7-like** | 605 | 1.63E-121 | 87.00% | C:integral to plasma membrane |
| **thioredoxin domain-containing protein 14 precursor** | 975 | 2.31E-72 | 94.30% | P:cell redox homeostasis |
| **phosphoribosylformylglycinamidine synthase-like** | 406 | 1.44E-80 | 79.40% | F:catalytic activity |
| **isoleucyl-trna cytoplasmic** | 3109 | 0 | 88.40% | C:cytosol |
| **protein rrp5 homolog** | 509 | 7.92E-74 | 70.30% | F:binding |
| **nucleolar protein 16-like** | 725 | 2.48E-47 | 82.90% | C:nucleolus |
| **vesicle-associated membrane protein-associated protein b c** | 402 | 2.49E-26 | 78.40% | F:protein dimerization activity |
| **alpha-methylacyl- racemase** | 600 | 3.47E-94 | 89.30% | P:metabolic process |
| **glucose regulated protein 75** | 286 | 3.02E-34 | 99.20% | P:protein folding |
| **eukaryotic translation initiation factor subunit 6** | 311 | 2.57E-65 | 99.20% | C:eukaryotic translation initiation factor 3 complex |
| **nadh dehydrogenase** | 597 | 9.59E-92 | 88.70% | P:response to oxidative stress |
| **methionine-trna synthetase** | 481 | 1.89E-10 | 84.33% | C:cytoplasm |
| **procollagen- 2-oxoglutarate 5-dioxygenase 3** | 415 | 6.21E-30 | 98.00% | F:procollagen glucosyltransferase activity |
| **rrna 2 -o-methyltransferase fibrillarin-like** | 1219 | 1.03E-154 | 97.60% | C:nucleolus |
| **s chain structural evidence for feedback activation by rasgtp of the ras-specific nucleotide exchange factor sos** | 304 | 2.15E-49 | 89.20% | P:axon guidance |
| **28s ribosomal protein mitochondrial precursor** | 545 | 2.56E-90 | 87.40% | C:mitochondrial large ribosomal subunit |
| **polyunsaturated fatty acid elongase** | 366 | 1.94E-44 | 93.00% | C:integral to membrane |
| **fatty acid elongase** | 294 | 2.59E-65 | 94.50% | C:integral to membrane |
| **5-methyltetrahydrofolate-homocysteine methyltransferase** | 1671 | 0 | 89.90% | P:tetrahydrofolate metabolic process |
| **phospholemman precursor** | 1152 | 1.98E-44 | 82.80% | C:membrane |
| **stress-70 mitochondrial precursor** | 753 | 1.04E-101 | 87.20% | P:protein folding |
| **stress-70 mitochondrial** | 1331 | 0 | 92.20% | P:protein folding |
| **d chain crystal structure of the pyruvate dehydrogenase component of human pyruvate dehydrogenase complex** | 512 | 1.79E-96 | 94.90% | F:pyruvate dehydrogenase (acetyl-transferring) activity |
| **60 kda heat shock mitochondrial** | 183 | 9.79E-34 | 100.00% | C:cell surface |
| **inosine-5 -monophosphate dehydrogenase 2** | 782 | 1.64E-93 | 96.10% | F:IMP dehydrogenase activity |
| **lrpprc protein** | 893 | 4.22E-162 | 84.60% | F:microtubule binding |
| **glutamate--cysteine ligase regulatory subunit** | 892 | 4.89E-133 | 79.20% | P:response to oxidative stress |
| **mitochondrial 28s ribosomal protein s21** | 1091 | 2.78E-47 | 91.10% | C:mitochondrial small ribosomal subunit |
| **isocitrate dehydrogenase** | 744 | 2.33E-169 | 97.30% | F:NAD or NADH binding |
| **zinc finger protein 706** | 795 | 2.57E-46 | 94.50% | F:zinc ion binding |
| **protein phosphatase regulatory subunit 2** | 447 | 1.06E-63 | 74.80% | F:phosphoprotein phosphatase inhibitor activity |
| **a chain tricyclic series of hsp90 inhibitors** | 159 | 4.83E-25 | 95.00% | P:axon guidance |
| **valyl-trna synthetase-like** | 508 | 2.62E-109 | 95.10% | P:valyl-tRNA aminoacylation |
| **nucleoplasmin-like protein no29** | 952 | 4.53E-49 | 84.40% | F:nucleic acid binding |
| **wd repeat domain 36** | 1742 | 0 | 79.80% | C:cytoplasm |
| **ptcd3 protein** | 840 | 3.60E-152 | 79.30% | C:mitochondrion |
| **hsp90-like protein** | 308 | 2.22E-59 | 97.40% | P:axon guidance |
| **vesicular integral-membrane protein vip36 precursor** | 803 | 8.58E-170 | 90.00% | C:membrane |
| **subfamily member 1** | 180 | 1.05E-32 | 93.80% | C:cytoplasm |
| **nodal modulator 1** | 1500 | 1.04E-165 | 88.30% | C:integral to membrane |
| **nadh dehydrogenase iron-sulfur protein mitochondrial precursor** | 412 | 5.13E-74 | 86.10% | P:photosynthesis, light reaction |
| **nogo-b receptor** | 675 | 6.61E-110 | 79.80% | F:receptor activity |
| **lim and sh3 domain protein 1** | 640 | 8.84E-14 | 94.90% | C:cytosol |
| **vacuolar proton pump subunit e 1** | 905 | 4.13E-86 | 89.50% | P:plasma membrane ATP synthesis coupled proton transport |
| **purine nucleoside phosphorylase-like** | 623 | 8.78E-34 | 87.30% | P:inosine catabolic process |
| **atp-dependent rna helicase ddx54** | 512 | 1.15E-57 | 75.00% | F:estrogen receptor binding |
| **116 kda u5 small nuclear ribonucleoprotein component isoform 3** | 1259 | 0 | 96.60% | F:translation elongation factor activity |
| **leucine-rich ppr motif-containing mitochondrial-like** | 726 | 1.31E-116 | 76.10% | P:transcription, DNA-dependent |
| **mki67 fha domain-interacting nucleolar phospho** | 571 | 1.22E-73 | 82.50% | C:nucleolus |
| **cytochrome c oxidase subunit mitochondrial precursor** | 756 | 6.91E-87 | 92.20% | P:embryonic organ development |
| **angiopoietin-related protein 1-like** | 1372 | 4.28E-174 | 72.90% | P:signal transduction |
| **nucleolar complex protein 4 homolog** | 337 | 1.31E-61 | 70.30% | C:nucleolus |
| **protein cdv3 homolog** | 1537 | 4.64E-64 | 76.90% | C:cytoplasm |
| **116 kda u5 small nuclear ribonucleoprotein component** | 1891 | 0 | 98.20% | P:RNA splicing |
| **thioredoxin-like protein 4a** | 494 | 3.42E-101 | 98.70% | C:spliceosomal complex |
| **protein canopy homolog 3-like** | 721 | 8.24E-113 | 88.20% | C:endoplasmic reticulum |
| **homocysteine-responsive endoplasmic reticulum-resident ubiquitin-like domain member 1 protein** | 269 | 1.19E-43 | 80.60% | F:protein binding |
| **gtp-binding protein 1** | 901 | 0 | 89.20% | F:GTP binding |
| **f-actin-capping protein subunit alpha-1** | 1004 | 0 | 92.20% | C:Z disc |
| **arginyl-trna cytoplasmic** | 185 | 6.89E-13 | 78.22% | C:cytoplasm |
| **glutamine-dependent nad(+) synthetase-like** | 526 | 2.89E-85 | 89.20% | F:NAD+ synthase activity |
| **proteasome assembly chaperone 4-like** | 470 | 2.48E-56 | 81.20% | C:proteasome complex |
| **elongation factor 1-delta** | 578 | 3.10E-60 | 75.10% | F:translation elongation factor activity |
| **dolichyl-diphosphooligosaccharide--protein glycosyltransferase subunit stt3b** | 1102 | 0 | 98.20% | P:protein amino acid N-linked glycosylation via asparagine |
| **cyclin-dependent kinase inhibitor 1** | 666 | 6.69E-65 | 61.50% | P:cellular response to stimulus |
| **39s ribosomal protein mitochondrial-like** | 557 | 4.85E-49 | 65.00% | C:ribonucleoprotein complex |
| **nuclear factor erythroid derived 2-like 1** | 634 | 2.00E-60 | 92.80% | P:heme biosynthetic process |
| **dna-directed rna polymerase ii subunit rpb4** | 514 | 3.16E-89 | 98.00% | F:DNA-directed RNA polymerase activity |
| **lyr motif-containing protein 7** | 214 | 1.42E-18 | 80.80% | C:mitochondrion |
| **dnaj homolog subfamily b member 1-like** | 548 | 2.33E-44 | 86.70% | P:protein folding |
| **dnaj homolog subfamily a member 1** | 1624 | 0 | 84.70% | F:heat shock protein binding |
| **eukaryotic translation initiation factor 3 subunit j** | 479 | 3.09E-65 | 91.50% | C:eukaryotic translation initiation factor 3 complex |
| **stt3b protein** | 470 | 9.25E-109 | 98.70% | P:protein amino acid N-linked glycosylation via asparagine |
| **-carotene 9 -oxygenase-like** | 351 | 9.52E-61 | 79.50% | F:oxidoreductase activity, acting on single donors with incorporation of molecular oxygen, incorporation of two atoms of oxygen |
| **6-phosphogluconolactonase** | 495 | 3.80E-68 | 82.00% | C:soluble fraction |
| **arginyl-trna cytoplasmic-like** | 265 | 3.67E-48 | 96.00% | C:cytoplasm |
| **elongation factor mitochondrial-like** | 1063 | 3.67E-164 | 78.50% | F:translation elongation factor activity |
| **40s ribosomal protein s27-like** | 242 | 2.14E-26 | 90.00% | P:activation of caspase activity |
| **cdc14 cell division cycle 14 homolog a ( cerevisiae)** | 1386 | 0 | 91.70% | F:protein tyrosine phosphatase activity |
| **methionyl-trna cytoplasmic** | 1422 | 0 | 79.60% | F:protein binding |
| **suppressor of g2 allele of skp1 homolog** | 667 | 8.76E-31 | 93.10% | F:binding |
| **bag family molecular chaperone regulator 3** | 252 | 1.20E-47 | 74.70% | P:apoptosis |
| **arginyl-trna synthetase** | 276 | 2.69E-45 | 93.50% | C:cytoplasm |
| **heat shock protein 4** | 385 | 1.81E-69 | 91.10% | C:cytosol |
| **monoamine oxidase** | 3759 | 0 | 90.00% | P:catecholamine metabolic process |
| **thioredoxin** | 704 | 4.98E-52 | 81.00% | P:cell redox homeostasis |
| **rrp15-like protein** | 1201 | 2.88E-50 | 73.00% | C:nucleolus |
| **gtp cyclohydrolase 1** | 808 | 2.62E-33 | 93.20% | P:protein heterooligomerization |
| **ependymin-1 precursor** | 934 | 5.67E-102 | 76.90% | C:extracellular region |
| **ester hydrolase c11orf54 homolog** | 2563 | 0 | 90.70% | F:hydrolase activity, acting on ester bonds |
| **cysteine-rich with egf-like domain protein 2 precursor** | 661 | 1.97E-151 | 83.40% | C:extracellular region |
| **nol6 protein** | 510 | 9.71E-78 | 75.70% | C:nucleolus |
| **transaldolase** | 1574 | 0 | 91.50% | C:soluble fraction |
| **ctp synthase 1** | 426 | 1.75E-86 | 94.20% | P:pyrimidine nucleotide biosynthetic process |
| **cytochrome b-c1 complex subunit mitochondrial precursor** | 496 | 2.52E-37 | 91.90% | P:mitochondrial electron transport, ubiquinol to cytochrome c |
| **x-box binding protein 1** | 324 | 1.31E-26 | 84.70% | F:transcription factor activity |
| **ap-2 complex subunit alpha-2-like isoform 2** | 2043 | 0 | 97.00% | F:binding |
| **golgi to er traffic protein 4 homolog** | 768 | 2.48E-150 | 95.20% |  |
| **phosphoribosylformylglycinamidine synthase** | 380 | 6.45E-53 | 89.40% | C:cytosol |
| **zgc:55605 protein** | 564 | 4.26E-83 | 88.90% | C:cytoplasm |
| **nucleolar protein 6-like** | 911 | 0 | 78.80% | C:nucleolus |
| **39s ribosomal protein mitochondrial precursor** | 676 | 1.06E-81 | 91.80% | C:ribosome |
| **cyclin-dependent kinase 2-associated protein 2** | 1049 | 2.81E-55 | 86.30% | F:kinase activity |
| **mitochondrial lon protease-like protein** | 515 | 1.04E-67 | 71.10% | P:response to hypoxia |
| **ras-related protein rap-1b precursor** | 797 | 1.57E-112 | 96.00% | C:cytosol |
| **diamine acetyltransferase 2-like** | 273 | 3.06E-40 | 86.40% | F:N-acetyltransferase activity |
| **transmembrane protein 49** | 620 | 1.29E-90 | 83.00% | C:integral to membrane |
| **alpha-2-macroglobulin receptor-associated** | 794 | 6.35E-164 | 83.90% | C:endoplasmic reticulum |
| **activator of 90 kda heat shock protein atpase homolog 1** | 1492 | 0 | 87.00% | C:cytoplasm |
| **phosphomevalonate kinase** | 714 | 3.07E-106 | 75.10% | C:cytoplasm |
| **heat shock 70 kda protein 4-like** | 4886 | 0 | 83.80% | F:ATP binding |
| **isoleucyl-trna synthetase** | 1430 | 0 | 91.10% | P:isoleucyl-tRNA aminoacylation |
| **novel protein beta-carotene oxygenase 2a** | 579 | 9.13E-105 | 85.10% | F:oxidoreductase activity, acting on single donors with incorporation of molecular oxygen, incorporation of two atoms of oxygen |
| **serine arginine-rich splicing factor 6 isoform 2** | 277 | 3.08E-44 | 99.10% | F:RNA binding |
| **ribosome biogenesis protein bop1** | 2130 | 0 | 87.80% | C:nucleoplasm |
| **dolichyl-p-man:man c -pp-dolichyl-alpha- -mannosyltransferase precursor** | 710 | 2.43E-129 | 85.50% | P:GPI anchor biosynthetic process |
| **novel protein vertebrate nardilysin (n-arginine dibasic convertase)** | 468 | 1.55E-97 | 89.40% | F:zinc ion binding |
| **pre-rrna-processing protein tsr1 homolog** | 1445 | 0 | 76.60% | C:nucleolus |
| **brix domain containing 2** | 1137 | 0 | 87.70% | C:nucleolus |
| **phosphatidylinositide phosphatase sac1-like** | 234 | 6.40E-45 | 92.90% | C:integral to endoplasmic reticulum membrane |
| **acyl carrier mitochondrial precursor** | 812 | 1.87E-82 | 85.00% | F:phosphopantetheine binding |
| **calnexin** | 1189 | 0 | 93.90% | P:protein secretion |
| **n-acetylated alpha-linked acidic dipeptidase-like 1** | 2557 | 0 | 75.60% | P:proteolysis |
| **small nuclear ribonucleoprotein sm d2** | 620 | 2.44E-64 | 99.20% | P:ncRNA metabolic process |
| **ruvb-like 1** | 322 | 9.99E-66 | 99.70% | P:histone H4 acetylation |
| **c11orf73 homolog** | 631 | 2.00E-39 | 91.80% | P:lung development |
| **cyclin-dependent kinase 2-interacting protein** | 694 | 4.61E-86 | 68.10% | F:protein binding |
| **e3 ubiquitin-protein ligase mdm2-like** | 366 | 3.52E-32 | 56.70% | C:intracellular |
| **ubiquinol-cytochrome c reductase complex chaperone cbp3 homolog** | 475 | 2.08E-102 | 87.30% | C:cytoplasmic membrane-bounded vesicle |
| **pyruvate dehydrogenase beta** | 466 | 6.24E-92 | 94.10% | P:oxidation reduction |
| **smooth muscle cell-specific protein sm22 alpha** | 1563 | 1.44E-126 | 93.10% | P:muscle organ development |
| **nadh dehydrogenase 1 alpha subcomplex subunit 6** | 479 | 1.63E-77 | 90.90% | C:mitochondrial inner membrane |
| **phosphatidylinositol 4-phosphate 3-kinase c2 domain-containing subunit gamma** | 580 | 9.81E-56 | 52.60% | C:intracellular membrane-bounded organelle |
| **thioredoxin reductase 3** | 1018 | 0 | 90.90% | P:cell redox homeostasis |
| **ribosomal rna processing protein 36 homolog** | 936 | 9.82E-58 | 79.20% | C:nucleolus |
| **nmda receptor-regulated protein 1** | 411 | 1.53E-60 | 94.00% | P:angiogenesis |
| **leucyl-trna cytoplasmic** | 2235 | 0 | 90.70% | F:leucine-tRNA ligase activity |
| **protein phosphatase catalytic subunit** | 2069 | 0 | 94.70% | C:cytosol |
| **eukaryotic translation initiation factor 6** | 646 | 1.32E-124 | 97.60% | C:cytoplasm |
| **valyl-trna synthetase** | 1027 | 1.38E-146 | 71.60% | C:cytoplasm |
| **vip36-like protein precursor** | 1387 | 1.99E-152 | 83.10% | C:endoplasmic reticulum membrane |
| **y-box binding protein** | 567 | 1.28E-63 | 76.10% | F:RNA binding |
| **pre-mrna-processing factor 19** | 2026 | 0 | 94.90% | P:protein polyubiquitination |
| **natural killer cell enhancement factor** | 986 | 1.72E-134 | 96.30% | P:cell redox homeostasis |

**Supplementary Table 2b**: Annotated genes matching **down**-regulated transfrags in the high temperature group of *M. duboulayi*. Mean similarity is computed as the average similarity value for all the hits of a given sequence.

| **Seq. Description** | **Seq. Length** | **min. eValue** | **mean Similarity** | **Primary Gene Ontology** |
| --- | --- | --- | --- | --- |
| **reverse transcriptase-like protein** | 399 | 1.23E-18 | 68.43% | F:RNA binding |
| **elmo domain containing 2** | 1922 | 9.20E-64 | 86.80% | C:cytoskeleton |
| **flavin-containing monooxygenase fmo gs-ox3-like** | 1198 | 3.12E-12 | 77.30% | F:monooxygenase activity |
| **transcription factor cp2-like protein 1** | 1469 | 2.90E-20 | 83.90% | P:viral genome replication |
| **transcription factor cp2-like 1** | 846 | 1.03E-109 | 78.60% | P:cellular developmental process |
| **glutathione s-transferase theta-1** | 1939 | 3.58E-16 | 86.90% | F:transferase activity |
| **glutamyl aminopeptidase** | 1434 | 3.86E-60 | 84.20% | P:angiogenesis |
| **novel protein glutamyl aminopeptidase (aminopeptidase a)** | 683 | 1.02E-41 | 79.60% | P:cell migration |
| **cytochrome p450** | 379 | 2.52E-25 | 90.30% | C:endoplasmic reticulum membrane |
| **tc1-like transposase** | 895 | 3.51E-11 | 59.00% | P:cellular macromolecule metabolic process |
| **nadh-cytochrome b5 reductase 2-like** | 374 | 3.88E-20 | 92.10% | F:cytochrome-b5 reductase activity |
| **sulfhydryl oxidase 1-like** | 1369 | 5.84E-156 | 71.60% | P:protein thiol-disulfide exchange |
| **sulfhydryl oxidase 1 isoform b precursor** | 809 | 3.22E-104 | 69.90% | P:protein thiol-disulfide exchange |
| **ovostatin homolog 1- partial** | 260 | 9.83E-07 | 78.80% | F:endopeptidase inhibitor activity |
| **mitochondrial uncoupling protein 1** | 678 | 5.98E-40 | 96.10% | P:mitochondrial transport |
| **uncoupling protein 4** | 1719 | 6.21E-56 | 96.90% | P:mitochondrial transport |
| **diphosphomevalonate decarboxylase** | 917 | 3.66E-13 | 86.80% | F:diphosphomevalonate decarboxylase activity |
| **mitogen-activated protein kinase 15** | 1581 | 0 | 62.60% | F:protein kinase activity |
| **complement component c3** | 901 | 2.14E-29 | 73.00% | C:extracellular region |
| **rna-directed dna polymerase from mobile element jockey-like** | 306 | 6.61E-27 | 79.30% | F:RNA binding |
| **ribosomal rna processing 1 homolog b ( cerevisiae)** | 630 | 6.63E-37 | 68.50% | C:nucleolus |
| **glyoxylate reductase hydroxypyruvate reductase-like** | 344 | 3.25E-43 | 87.90% | F:oxidoreductase activity, acting on the CH-OH group of donors, NAD or NADP as acceptor |
| **chromodomain helicase dna binding protein isoform cra_a** | 3060 | 0 | 86.20% | P:regulation of transcription from RNA polymerase II promoter |
| **protein fam13a-like** | 807 | 1.77E-34 | 91.60% | C:cytosol |
| **c-type lectin** | 1545 | 1.10E-19 | 80.20% | F:sugar binding |
| **plakophilin 3** | 2765 | 0 | 73.90% | F:binding |
| **glucokinase** | 389 | 9.60E-74 | 96.10% | P:positive regulation of glycogen biosynthetic process |
| **glucokinase (hexokinase maturity onset diabetes of the young 2)** | 365 | 1.37E-71 | 93.60% | P:positive regulation of glycogen biosynthetic process |
| **hexokinase ii** | 653 | 2.43E-106 | 65.10% | P:transmembrane transport |
| **pancreatic progenitor cell differentiation and proliferation factor b** | 508 | 5.37E-09 | 79.00% | P:multicellular organismal development |
| **lysosome membrane protein 2-like** | 854 | 8.71E-15 | 85.30% | C:membrane |
| **ethanolamine kinase 1** | 2775 | 5.00E-71 | 89.50% | C:cytoplasm |
| **thyrotrophic embryonic factor** | 1835 | 9.16E-157 | 75.60% | P:cellular response to light stimulus |
| **6-pyruvoyl tetrahydrobiopterin synthase** | 1775 | 1.01E-84 | 87.70% | F:6-pyruvoyltetrahydropterin synthase activity |
| **serine--pyruvate mitochondrial precursor** | 513 | 2.50E-25 | 87.50% | P:metabolic process |
| **catechol-o-methyltransferase domain-containing protein 1** | 673 | 1.45E-75 | 85.30% | F:O-methyltransferase activity |
| **novel methyltransferase protein** | 801 | 3.03E-10 | 85.33% | F:O-methyltransferase activity |
| **purine nucleoside phosphorylase** | 1365 | 1.20E-10 | 88.60% | F:purine-nucleoside phosphorylase activity |
| **phosphoethanolamine n-methyltransferase 3-like** | 1708 | 9.61E-16 | 85.40% | F:phosphoethanolamine N-methyltransferase activity |
| **nuclear receptor coactivator 7-like** | 544 | 6.73E-60 | 82.30% | F:receptor activity |
| **phosphatidate phosphatase lpin1-like** | 448 | 1.98E-88 | 94.10% | P:mitochondrial fission |
| **lipin 1** | 1031 | 3.06E-170 | 87.30% | P:negative regulation of transcription from RNA polymerase II promoter |
| **pol polyprotein** | 3049 | 2.35E-92 | 61.70% | F:binding |
| **ribosomal protein l22-like 1** | 514 | 6.78E-53 | 94.60% | C:ribosome |
| **leukocyte cell-derived chemotaxin 2** | 550 | 3.64E-32 | 62.40% | P:response to stimulus |
| **sh3 and px domain-containing protein 2b** | 1658 | 0 | 83.40% | F:phosphatidylinositol-5-phosphate binding |
| **nuclear receptor corepressor 2-like** | 1292 | 2.36E-141 | 67.70% | P:heart morphogenesis |
| **claudin 26** | 2043 | 1.04E-20 | 73.30% | C:integral to membrane |
| **equilibrative nucleoside transporter 1-like** | 1438 | 1.13E-94 | 80.50% | C:membrane |
| **histamine n-methyltransferase** | 1015 | 1.19E-130 | 70.90% | P:respiratory gaseous exchange |
| **trans- -dihydrobenzene- -diol dehydrogenase** | 445 | 1.62E-78 | 85.60% | P:oxidation reduction |
| **udp-glucuronosyltransferase 2a2-like isoform 2** | 664 | 1.10E-93 | 79.10% | F:transferase activity, transferring hexosyl groups |
| **gag-pol precursor polyprotein** | 2308 | 1.40E-46 | 52.80% | P:cellular process |
| **pr gag-pro-pol** | 4332 | 0 | 50.10% | F:binding |
| **period homolog 3** | 2183 | 1.01E-173 | 68.10% | C:cytoplasm |
| **peroxisome proliferator-activated receptor alpha** | 845 | 2.04E-78 | 96.80% | P:steroid hormone mediated signaling pathway |
| **nuclear receptor subfamily 1 group d member 2** | 1627 | 3.54E-102 | 90.20% | P:steroid hormone mediated signaling pathway |
| **solute carrier family facilitated glucose transporter member 9-like** | 1999 | 0 | 82.20% | F:glucose transmembrane transporter activity |
| **erythroid protein** | 1342 | 0 | 82.70% | C:cytoskeleton |
| **plexin a2** | 960 | 6.34E-142 | 84.50% | P:axon guidance |
| **low quality protein: plexin-a2-like** | 5485 | 0 | 95.60% | P:axon guidance |
| **cholesteryl ester transfer** | 1318 | 0 | 68.00% | P:lipid transport |
| **lanosterol synthase** | 365 | 6.18E-15 | 79.50% | F:intramolecular transferase activity |
| **55 kda erythrocyte membrane protein** | 500 | 2.00E-91 | 86.20% | C:intracellular non-membrane-bounded organelle |
| **probable atp-dependent rna helicase ddx5-like** | 1037 | 1.47E-35 | 97.70% | F:transcription cofactor activity |
| **high affinity nerve growth factor receptor-like** | 504 | 2.28E-90 | 77.70% | P:Ras protein signal transduction |
| **s100 calcium binding protein p** | 502 | 6.93E-19 | 77.20% | P:endothelial cell migration |
| **tubulin polyglutamylase complex subunit 2** | 1659 | 4.79E-28 | 80.20% | C:cytoskeleton |
| **myelin transcription factor 1-like** | 1332 | 1.26E-85 | 94.80% | P:cell differentiation |
| **acyl- synthetase family member mitochondrial-like** | 1449 | 0 | 79.20% | F:ligase activity |
| **hepatocyte growth factor-like protein** | 1535 | 0 | 85.00% | F:serine-type endopeptidase activity |
| **bifunctional protein ncoat-like** | 2947 | 2.09E-142 | 92.10% | P:positive regulation of insulin secretion |
| **protein shisa-2-like** | 420 | 1.10E-59 | 73.30% | C:membrane |
| **rasgap-activating-like protein 1** | 947 | 4.65E-83 | 88.20% | F:Ras GTPase activator activity |
| **acyl- synthetase long-chain family member 4** | 911 | 2.74E-62 | 89.50% | P:long-chain fatty-acyl-CoA biosynthetic process |
| **bone morphogenetic protein 1-like** | 1373 | 0 | 94.40% | P:determination of ventral identity |
| **zinc finger protein 521** | 1424 | 0 | 87.10% | P:cell differentiation |
| **cbp p300-interacting transactivator 2** | 1615 | 2.99E-43 | 72.30% | C:nucleus |
| **band 3 anion exchange protein** | 1357 | 0 | 89.80% | F:inorganic anion exchanger activity |
| **histone-lysine n-methyltransferase setd3-like** | 1683 | 3.00E-148 | 88.50% | P:peptidyl-lysine monomethylation |
| **transposable element tc1 transposase** | 3936 | 9.27E-33 | 53.00% | P:cellular macromolecule metabolic process |
| **f-box only protein 3** | 2599 | 1.55E-114 | 85.60% | F:ubiquitin-protein ligase activity |
| **sorting nexin-14** | 1531 | 0 | 91.40% | P:cell communication |
| **cohesin subunit sa-1** | 493 | 2.35E-42 | 93.30% | C:chromatin |
| **72 kda type iv collagenase precursor** | 1248 | 0 | 91.20% | C:collagen |
| **achain x-ray structure of beta catenin in complex with bcl9** | 393 | 3.72E-51 | 97.30% | F:estrogen receptor binding |
| **lysophosphatidylcholine acyltransferase 1-like** | 1464 | 6.81E-173 | 74.00% | F:1-acylglycerophosphocholine O-acyltransferase activity |
| **glucose-fructose oxidoreductase domain-containing protein 1-like** | 2451 | 4.30E-38 | 97.70% | C:extracellular region |
| **glutamate receptor 3-like isoform 2** | 795 | 2.38E-171 | 98.50% | C:membrane fraction |
| **fh2 domain-containing protein 1-like** | 303 | 4.23E-44 | 82.20% | P:cellular component organization |
| **5- partial** | 1631 | 0 | 85.70% | F:5-oxoprolinase (ATP-hydrolyzing) activity |
| **inositol -trisphosphate receptor type 1** | 405 | 1.40E-72 | 89.90% | P:nerve growth factor receptor signaling pathway |
| **g2 m phase-specific e3 ubiquitin-protein ligase** | 495 | 1.60E-63 | 71.50% | C:intracellular membrane-bounded organelle |
| **mitogen-activated protein kinase kinase kinase kinase 5-like** | 708 | 9.46E-129 | 70.40% | P:response to stimulus |
| **high mobility group protein partial** | 642 | 7.29E-87 | 93.30% | F:transcription factor activity |
| **high mobility group protein b1** | 560 | 5.95E-73 | 94.60% | F:transcription factor activity |
| **myosin regulatory light chain atrial isoform** | 535 | 8.80E-83 | 90.40% | P:sarcomere organization |
| **matrix metalloproteinase 2** | 332 | 9.97E-73 | 96.20% | C:collagen |
| **nei endonuclease viii-like 1** | 807 | 6.56E-92 | 74.50% | C:cytoplasm |
| **svil protein** | 698 | 1.08E-28 | 84.90% | C:podosome |
| **svil partial** | 1559 | 0 | 74.10% | C:costamere |
| **synaptotagmin i** | 1106 | 0 | 92.60% | C:cell junction |
| **5 -amp-activated protein kinase subunit gamma-2** | 548 | 3.75E-45 | 61.90% | F:cAMP-dependent protein kinase regulator activity |
| **polycomb protein suz12-like** | 1810 | 1.36E-39 | 69.60% | P:negative regulation of transcription from RNA polymerase II promoter |
| **transposable element tcb1 transposase** | 927 | 1.18E-24 | 55.20% | P:cellular macromolecule metabolic process |
| **actin-binding lim protein 1 long isoform isoform cra_a** | 1211 | 4.22E-35 | 95.40% | P:axon guidance |
| **trinucleotide repeat containing 4** | 1146 | 3.54E-83 | 98.90% | C:cytoplasm |
| **l _3** | 417 | 3.11E-24 | 60.70% | F:binding |
| **extracellular sulfatase sulf-1** | 745 | 2.14E-144 | 81.10% | P:negative regulation of fibroblast growth factor receptor signaling pathway |
| **cytochrome p450 1a** | 1058 | 5.02E-152 | 91.50% | C:endoplasmic reticulum membrane |
| **cytochrome p450 1a1** | 602 | 2.87E-94 | 96.30% | C:endoplasmic reticulum membrane |
| **cytochrome p450 partial** | 453 | 3.76E-93 | 95.80% | C:endoplasmic reticulum membrane |
| **trinucleotide repeat containing 4 isoform cra_a** | 943 | 5.47E-59 | 97.40% | C:cytoplasm |
| **sh3 and px domain-containing protein 2b-like** | 1259 | 3.97E-89 | 79.50% | F:phosphatidylinositol-5-phosphate binding |
| **serpin peptidase clade a (alpha-1 antitrypsin) member 7** | 979 | 9.69E-33 | 78.90% | F:serine-type endopeptidase inhibitor activity |
| **neuronal cell adhesion molecule short isoform** | 383 | 3.58E-14 | 93.40% | P:axon guidance |
| **egf-like repeat and discoidin i-like domain-containing protein 3-like** | 996 | 0 | 84.60% | P:cell adhesion |
| **5-aminolevulinate erythroid- mitochondrial-like** | 2087 | 0 | 83.70% | P:response to hypoxia |
| **ras-related protein rab-13-like** | 1890 | 1.31E-67 | 91.10% | P:vesicle-mediated transport |
| **wd repeat domain phosphoinositide-interacting protein 4** | 726 | 7.57E-124 | 96.00% | P:response to starvation |
| **heme oxygenase** | 324 | 5.95E-61 | 91.80% | C:microsome |
| **ras and ef-hand domain-containing** | 1103 | 4.06E-91 | 71.70% | F:nucleotide binding |
| **synaptobrevin homolog ykt6** | 1428 | 3.21E-121 | 94.00% | C:Golgi membrane |
| **ectonucleotide pyrophosphatase phosphodiesterase family member 2-like** | 555 | 1.92E-113 | 86.10% | F:zinc ion binding |
| **protein creg2-like** | 952 | 9.34E-142 | 78.60% | C:cytoplasmic part |
| **ras-related protein rab-30** | 2110 | 3.72E-133 | 94.80% | P:small GTPase mediated signal transduction |
| **tyrosine-protein kinase mer-like** | 1383 | 5.95E-32 | 82.80% | P:platelet activation |
| **collagen alpha-1 chain-like** | 1366 | 9.09E-153 | 77.00% | P:proteoglycan metabolic process |
| **cat eye syndrome chromosome candidate 5 homolog** | 852 | 2.79E-171 | 85.20% | P:metabolic process |
| **lysosomal alpha-glucosidase-like** | 2886 | 0 | 81.50% | F:carbohydrate binding |
| **vitellogenin b** | 2359 | 0 | 88.10% | F:lipid transporter activity |
| **vitellogenin ab** | 2825 | 0 | 84.90% | F:lipid transporter activity |
| **c-jun-amino-terminal kinase-interacting protein 4 isoform partial** | 1599 | 0 | 57.60% | F:protein binding |
| **ring finger protein 213-like** | 714 | 1.69E-106 | 70.60% | F:nucleoside-triphosphatase activity |
| **guanine nucleotide-binding protein g subunit alpha** | 981 | 0 | 97.60% | F:signal transducer activity |
| **autosomal dominant 5** | 973 | 2.49E-56 | 48.00% | P:regulation of biosynthetic process |
| **e3 ubiquitin-protein ligase nrdp1-like** | 482 | 5.26E-54 | 78.50% | F:ubiquitin-protein ligase activity |
| **vomeronasal type-2 receptor 1-like** | 370 | 1.50E-22 | 78.10% | F:RNA-directed DNA polymerase activity |
| **type ii** | 386 | 6.23E-56 | 88.60% | P:arginine metabolic process |
| **supervillin isoform cra_b** | 852 | 1.61E-137 | 68.40% | C:costamere |
| **c-type natriuretic peptide 2 precursor** | 368 | 5.98E-07 | 90.50% | C:extracellular region |
| **replicase polyprotein 1ab** | 1039 | 2.01E-20 | 47.00% | F:hydrolase activity |
| **ubl carboxyl-terminal hydrolase 18-like** | 1035 | 4.81E-94 | 63.70% | F:peptidase activity |
| **protein hook homolog 1** | 3964 | 0 | 85.00% | C:FHF complex |
| **novel protein vertebrate vitrin** | 498 | 6.32E-50 | 80.30% | F:glycosaminoglycan binding |
| **erythrocyte membrane protein band (elliptocytosis rh-linked)** | 1416 | 1.61E-83 | 50.00% | F:binding |
| **thymidine phosphorylase** | 1676 | 0 | 75.20% | C:cytosol |
| **e3 ubiquitin-protein ligase march1-like** | 866 | 3.21E-116 | 89.20% | C:cytoplasmic vesicle membrane |
| **f-box lrr-repeat protein 2** | 382 | 4.87E-82 | 90.00% | C:membrane |
| **dual specificity tyrosine-phosphorylation-regulated kinase 1b** | 1000 | 0 | 97.60% | P:protein amino acid autophosphorylation |
| **transmembrane protein 192** | 2636 | 6.58E-112 | 69.70% | C:membrane |
| **an1-type zinc finger protein 3** | 1217 | 1.41E-11 | 89.50% | F:zinc ion binding |
| **pyruvate dehydrogenase isoenzyme 2** | 3204 | 0 | 89.90% | P:two-component signal transduction system (phosphorelay) |
| **nad h quinone 1** | 670 | 3.68E-126 | 76.10% | F:cytochrome-b5 reductase activity |
| **coagulation factor vii** | 770 | 3.02E-130 | 68.70% | F:calcium ion binding |
| **coagulation factor vii precursor** | 984 | 5.07E-154 | 69.00% | F:calcium ion binding |
| **next to brca1 gene 1** | 625 | 7.48E-111 | 68.60% | P:macroautophagy |
| **protein fam57a** | 1045 | 9.07E-145 | 72.70% | C:integral to membrane |
| **cbp p300-interacting transactivator 3b** | 2143 | 5.19E-44 | 89.10% | C:nucleus |
| **glutamate decarboxylase-like protein 1** | 2021 | 0 | 84.60% | F:pyridoxal phosphate binding |
| **phytanoyl- peroxisomal-like** | 1589 | 0 | 87.00% | F:metal ion binding |
| **scinderin like a** | 2695 | 0 | 86.50% | P:eye development |
| **beta-2 microglobulin** | 1079 | 1.01E-48 | 83.60% | P:immune response |
